# Neutralizing antibody titers to Omicron six months after vaccination with BNT162b2 in Colombia

**DOI:** 10.3389/fimmu.2022.1102384

**Published:** 2022-12-21

**Authors:** María I. Zapata-Cardona, Lizdany Flórez-Álvarez, Tulio J. Lopera, Mateo Chvatal-Medina, Wildeman Zapata-Builes, Francisco J. Diaz, Wbeimar Aguilar-Jimenez, Natalia Taborda, Juan C. Hernandez, Maria T. Rugeles

**Affiliations:** ^1^ Grupo Inmunovirología, Facultad de Medicina, Universidad de Antioquia UdeA, Medellín, Colombia; ^2^ Institute of Biomedical Sciences, University of São Paulo, São Paulo, Brazil; ^3^ Infettare, Facultad de Medicina, Universidad Cooperativa de Colombia., Medellín, Colombia; ^4^ Grupo de Investigaciones Biomédicas Uniremington, Programa de Medicina, Facultad de Ciencias de la Salud, Corporación Universitaria Remington, Medellín, Colombia

**Keywords:** SARS-CoV-2, vaccine, neutralizing antibodies, omicron, COVID-19

## Abstract

The emergence of the Omicron variant has generated concerns about the efficacy of COVID-19 vaccines. We evaluated the serum neutralizing activity of antibodies against the Omicron (lineage BA.1.1) by plaque reduction neutralizing test, as well as its correlation with age and gender, in a Colombian cohort six months after being vaccinated with BNT162b2 (Pfizer/BioNTech). Compared to all other variants analyzed, a significantly lower neutralizing activity (p<0.001) was observed against Omicron. Interestingly, older individuals exhibited lower titers against Omicron than those younger than 40. No statistical differences in neutralizing activity were observed according to gender. Our results showed that two doses of BNT162b2 might not provide robust protection against the Omicron variant over time. It is necessary to consider including changes in the composition of the vaccines to protect against new emerging variants of SARS-CoV-2 and campaigns to implement additional booster vaccinations.

## 1 Introduction

Different variants of SARS-CoV-2 (severe acute respiratory syndrome coronavirus 2) have been identified since the beginning of the COVID-19 pandemic. Some have been designated as variants of concern (VOC), including Omicron (1). This variant (B.1.1.529 lineage) was reported for the first time in Botswana and South Africa (1). Omicron and its sub-lineages have become the dominant circulating strains worldwide, causing an increase in reported cases of COVID-19 at the end of 2022, especially in Japan and South Korea (2, 3). This VOC has been associated with a ttenuated pathogenicity (4),increased transmissibility, and a greater magnitude of breakthrough infections and reinfections due to distinctive immune evasion mechanisms (1, 4–6).

Compared to the reference genome reported in Wuhan, Omicron has approximately fifty mutations, most located in the Spike (S) protein (7, 8). This protein interacts with the human ACE2 receptor to enter into cells, participates in the fusion of viral envelope and cellular membranes, and is the main viral target of neutralizing antibodies produced in vaccinated individuals and convalescent COVID-19 patients (7, 9). Recent studies have indicated that mutations in Omicron Spike at or near the furin-like cleavage site (T547K, D614G, H655Y, N679K, and P681H) or in S2 (N764K, D796Y, N856K, Q954H, N969K, and L981F) may be related to reduced efficiency in proteolytic cleavage by host proteases, affecting the viral pathogenesis (10–13). However, detailed studies are needed for a better understanding of the pathogenesis and virulence of this variant.

On the other hand, amino acid mutations in the RBD (receptor binding domain) of Omicron Spike, including G339D, S371L, S373P, S375F, K417N, N440K, G446S, S477N, T478K, E484A, Q493R, G496S, Q498R, N501Y, Y505H, and other in the NTD (N-terminal domain) such as A67V, del69-70, T95I, G142D, del143-145, N211I, del212, and ins214EPE, have been associated with increased ACE2 binding affinity and evasion of the humoral response generated by infection or vaccination (9, 12, 14, 15).

Consequently, the new SARS-CoV-2 variants represent a challenge for COVID-19 vaccines that were designed to recognize the S protein of the ancestral virus, including those using mRNA, protein, and viral vector platforms (16, 17). Concerning BNT162b2 (Pfizer/BioNTech), an mRNA vaccine, previous studies have reported a reduction of neutralizing capacity over time against SARS-CoV-2 variants that have circulated worldwide, especially against Delta and Mu (18–20). Considering this evidence and that immunity against SARS-CoV-2 is highly variable depending on the population characteristics (21), the efficacy of this vaccine against Omicron needs to be evaluated in different places worldwide. In this study, we measured the serum neutralizing activity against the Omicron variant (lineage BA.1.1) six months post-vaccination with BNT162b2 in a Colombian cohort and compared these results with neutralization titers for B.1, Gamma, Alpha, Delta and Mu variants, previously reported (18). In addition, we evaluated the neutralization titers to the Omicron variant and correlated them with the age and gender of the donors.

## 2 Materials and methods

### 2.1 Study design, ethical considerations, and samples collection

A cross-sectional cohort study was conducted with sixty BNT162b2 (Pfizer/BioNTech) fully vaccinated Colombian donors. All individuals received the BNT162b2 vaccine in a double-dose scheme, with an inter-dose interval of three weeks, per the interim recommendations issued by the WHO. Basic demographic information, including age, sex, and any relevant COVID-19 history, was obtained from each participant. Eligibility and exclusion criteria were described in a previous study derived from the same project (18).

The study was designed and conducted following the Declaration of Helsinki and Colombian legislation and was approved by the Ethics Committee of the Universidad de Antioquia (# 006/2021). After thoroughly explaining the project, all subjects signed a written informed consent and provided blood samples.

Peripheral blood samples of each voluntary were collected 180 days after receiving the second vaccination dose (with a window of ± 28 days). All the samples were taken in non-anticoagulant tubes (dry tubes) and were centrifuged for 8min at 1700rpm. Then the serum aliquots were stored at -80°C until their use. The heat-inactivation of samples was performed as described in the previously published article (18) at 56°C for 30 min.

### 2.2 Virus

SARS-CoV-2 variant Omicron (BA.1.1, EPI_ISL_8374770) was isolated from a Colombian donor. In addition, the results were compared with the neutralizing antibodies titers against ancestral strain (lineage: B.1, ID accession: EPI_ISL_536399), Gamma (P.1, EPI_ISL_4926393), Alpha (B.1.117, EPI_ISL_4549188), Delta (B.1.617.2, EPI_ISL_5103929), and Mu (B.1.621, EPI_ISL_4005445) (18).

### 2.3 Neutralizing assay

Neutralizing activity of serum samples against the Omicron variant (BA.1.1 lineage) was detected by a 50% plaque reduction neutralization test (PRNT50) as previously described (18). Briefly, Vero E6 cells were cultured with serial dilutions of heat-inactivated serum samples and each SARS-CoV-2 variant for 1h at 37°C, 5% CO_2_. For the Omicron variant, the inoculum was removed, and 15 µg/mL of porcine trypsin-EDTA (Sigma) was added (200 µL per well) for 1h at 37°C, 5% CO_2_. The monolayers were washed with PBS. Then 1 mL of the semisolid medium (1.75% carboxymethylcellulose, 2% FBS, 1% penicillin-streptomycin, and DMEM 1X) was added. Cells were incubated at 37°C for 4-5 days. Finally, the monolayers were fixed and stained with 4% formaldehyde and 2% crystal violet, respectively. A 50% reduction in plaque with count respect to the infection control was defined as the neutralization endpoint. Neutralizing antibodies titer is reported as the calculated reciprocal dilution.

### 2.4 Statistical analysis

All data were analyzed using GraphPad Prism software, version 8.0 (California, USA). The Wilcoxon test for paired samples was used to compare the neutralization titer between Omicron variant and the ancestral B.1. Spearman’s rank correlation coefficient was used to determine correlation of age with neutralization titer against the Omicron variant. A p-value <0.05 in these tests was considered statistically significant.

## 3 Results

**Figure 1:**
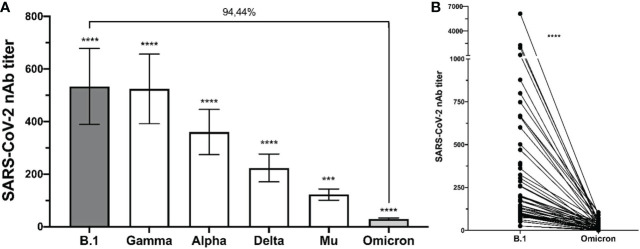
Decrease in nAbs against Omicron in comparison to other variants. The comparison between the neutralizing antibody titers of the individuals after six months of being fully vaccinated is shown. **(A)** bulk results; the statistical analysis was performed using Kruskal-Wallis test with a confidence level of 95% and *post hoc* tests (or multiple benchmarks) HDS of Dunn. **** p<0.0001, *** p<0.001 for each variant compared with the Omicron. **(B)** individual results comparing B.1 and Omicron variant; the statistical analysis was performed by Wilcoxon’s test.

The sociodemographic characteristics of the participants were previously described (18). Briefly, 16.6% of individuals reported comorbidities, where arterial hypertension and diabetes were the most prevalent (5% and 3.33%, respectively) (18). The PRNT in all BNT162b2-vaccinated individuals against Omicron was lower than the values previously obtained for other SARS-CoV-2 variants (18) (p<0.001) at six months post-vaccination (**Figure 1**). A reduction in the neutralizing titer rate of 94,44% was obtained for the Omicron variant compared to the ancestral strain B.1 (**Figures 1A, B**).

**Figure 2:**
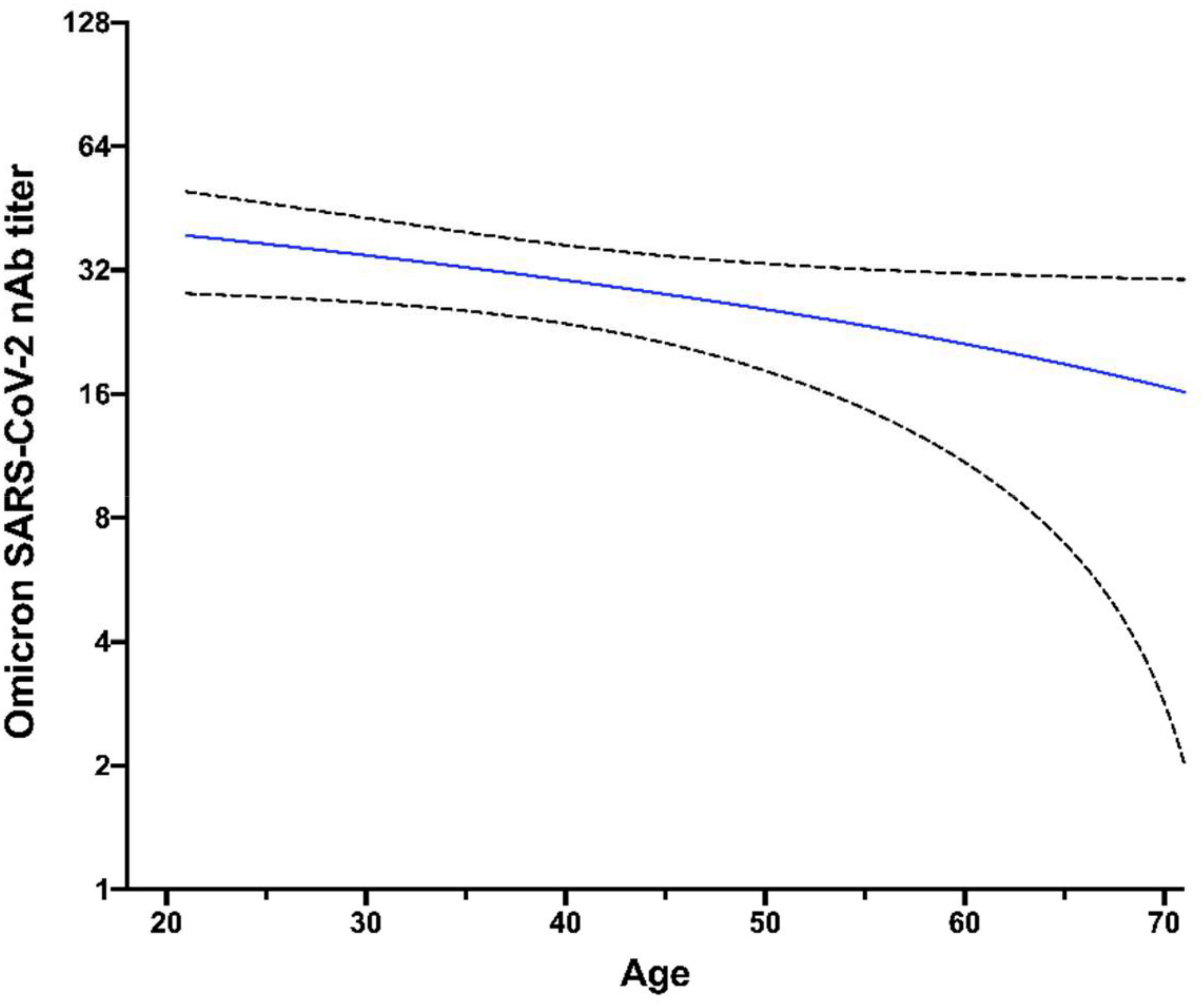
Lower NAbs titer against Omicron variant in older donors. The distribution of neutralizing antibody (NAbs) titers after six months of completing the vaccination scheme is shown for the Omicron variant, according to the age distribution of studied individuals. The statistical analysis was performed using Spearman’s correlation. Dashed lines indicate the confidence interval of data and the continuous line indicates the correlation (average of nAbs titers according to the age of participants).

**Figure 3:**
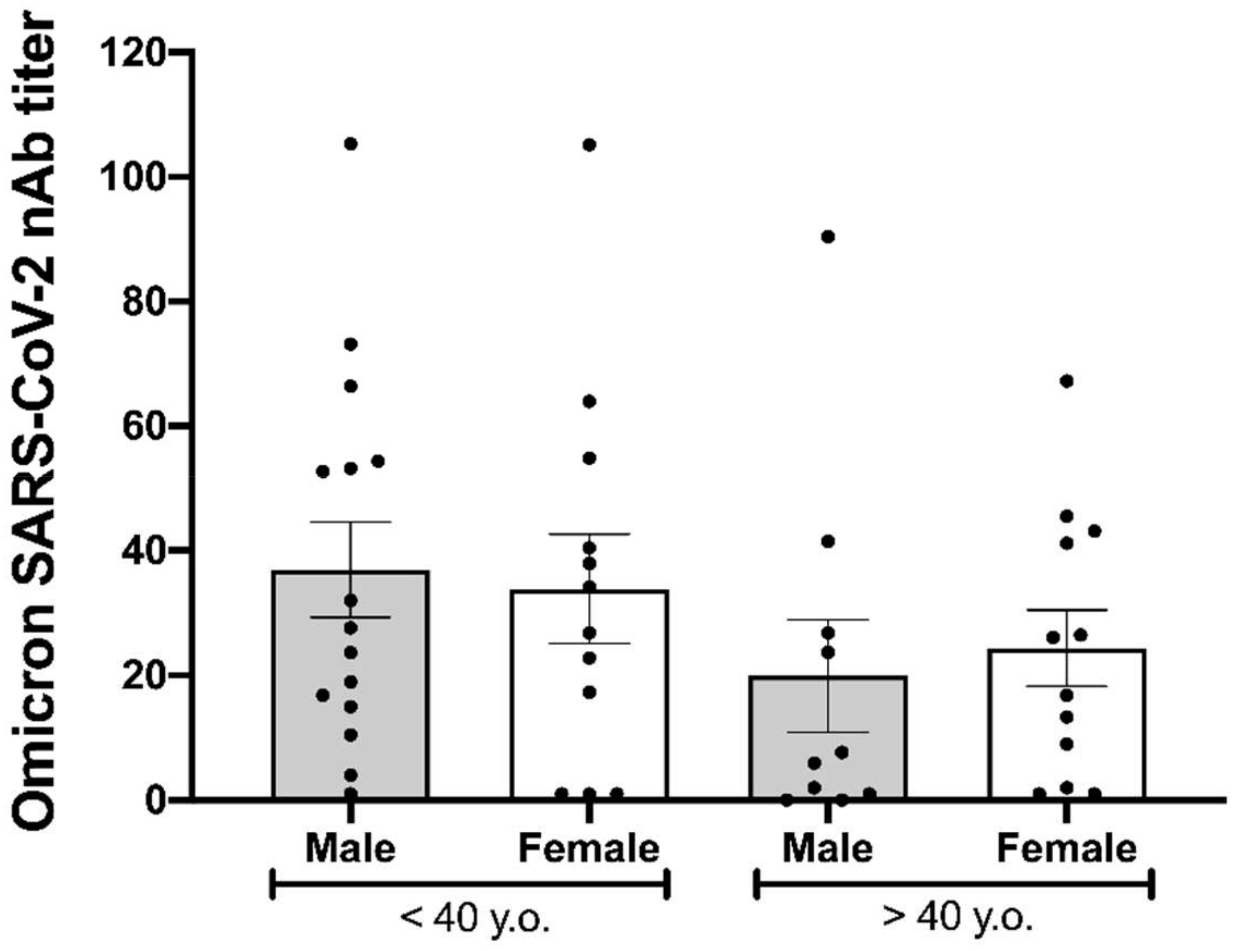
Immunogenicity of BNT162b2 at 180 days after being fully vaccinated. The neutralizing antibody (NAb) titers against the Omicron variant six months after being fully vaccinated according to age over or under 40 years and gender. Statistical comparison was made using the Kruskal-Wallis test with a confidence level of 95% and *post hoc* tests (or multiple benchmarks) HDS of Dunn were applied.

In addition, we analyzed neutralization titers to the Omicron variant according to the age and gender of participants. As shown in **Figure 2**, the neutralizing titer against the Omicron variant was negatively correlated with the age of donors (r= -0.029, p= 0.046). However, no differences in the neutralizing titers were observed according to the gender of the participants (**Figure 3**).

## 4 Discussion

The emergence and rapid spread of the Omicron variant globally have raised concerns regarding if preventive interventions, such as vaccination, would be ineffective against this variant (22). Here, we compared the neutralizing activity of serum from BNT162b2-vaccinated individuals against the Omicron (BA.1.1) variant compared to the ancestral strain (B.1), as well as Gamma, Alpha, Delta and Mu variants, finding that Omicron exhibited significantly more neutralization resistance (p < 0.0001), respect to all variants evaluated. Similar to our results, several studies worldwide have reported a reduction in PRNT against the Omicron variant compared to ancestral SARS-CoV-2 in individuals vaccinated with BNT162b2 (23–25). Zeng et al. found that the Omicron variant exhibited significantly more neutralization resistance than B.1, Alpha, Beta, and Delta variants after two doses of Pfizer/BioNTech vaccination (26). Furthermore, another study reported that neutralization titers to Omicron fell substantially between 28 days and 6 months after the second dose of the BNT162b2 vaccine (27).

We also analyzed the relation between the humoral immune response induced by BNT162b2 against the Omicron variant and the demographic characteristics of vaccinated individuals, such as age and gender. We found that neutralizing antibody titers against Omicron 6 months post-vaccination decreases as age increases, as previously reported (28, 29). Although this negative correlation was moderate, it could be due to the declining quality of the humoral immune response according to age, as aged B cells exhibit less potential for somatic hypermutation, which may affect the generation of robust neutralizing antibody titers (30). In contrast, our results did not show significant differences in the neutralizing capacity against Omicron between the males and females within each age group. This finding agrees with Dörschug et al. (31), who monitored the humoral response induced by BNT162b2 using a spike protein-based IgG serological immunoassay without finding significant differences by gender.

Concerning different sub-lineages of Omicron, Hachmann et al. found that six months after the two BNT162b2 doses, neutralizing antibody titers against BA.1, BA.2, BA.2.12.1, and BA.4, or BA.5 were lower than those against the reference SARS-CoV-2 (WA1/2020) (32). Moreover, humoral responses against the BA.4, BA.5, and BA.2.12.1 sub-lineages were lower than the response against BA.1 and BA.2 (32), suggesting that the new sub-lineages of the Omicron variant could have a higher ability to escape neutralizing antibodies. Despite this evidence, different studies have suggested that the booster vaccine dose could amplify neutralizing antibodies targeted to conserved epitopes on S protein or increase the affinity of existing neutralizing antibodies against the SARS-CoV-2 variants, including those against different Omicron sub-lineages (22, 33, 34).

Regarding homologous boosters, a study conducted in France found a substantial increase in the neutralization activity against Omicron (half maximal effective dilution of 722) one month after the BNT162b2 third dose, compared to samples collected five months after two-dose vaccination (33). On the other hand, Hoffmann et al. reported that the neutralization efficacy of the Omicron S was less than to B.1 and Delta (8-fold and 2-fold, respectively) after vaccination with three doses of BNT162b2 (22). In addition, Bar-On et al. reported that a third immunization with BNT162b2 reduced the rates of confirmed infection and severe illness by 11.3-fold and 19.5-fold, respectively, concerning non-booster individuals (35). Concerning heterologous vaccination, a study conducted in Singapore found that individuals BNT162b2-vaccinated who had received a booster with mRNA-1273 had higher neutralizing antibody levels against the Omicron variant (inhibition percentage of 84.3%) than the individuals with homologous booster (72.8%) after 28 days post- third dose (36). These findings suggest that homologous and heterologous boosting might improve the humoral immune response against the Omicron variant compared to two doses of BNT162b2, and generate a protection against severe forms of the disease.

Unfortunately, our study did not include the measuring of neutralizing titers against Omicron in individuals after the third dose of vaccination (booster), limiting the scope of our results. Besides, the quantification of T- cell responses was not determined.

The data presented in this study showed the impact of Omicron on *in vitro* neutralizing activity of subjects 6 months post-vaccination with two doses of BNT162b2 (Pfizer/BioNTech). Additionally, studies evaluating the effectiveness of COVID-19 vaccines against the new Omicron sub-lineages and each emerging variant of SARS-CoV-2 are needed. Cohorts with a history of infection and booster vaccination exposure should also be studied.

## Data availability statement

The original contributions presented in the study are included in the article/Supplementary Material. Further inquiries can be directed to the corresponding author.

## Ethics statement

The studies involving human participants were reviewed and approved by Ethics Committee of the Universidad de Antioquia (# 006/2021). The patients/participants provided their written informed consent to participate in this study.

## Author contributions

MZ-C: Design, Neutralization assays, Formal analysis, Writing – Original Draft; MC-M, TL, LF-A: Subjects recruitment, Experiments, Formal analysis; WZ-B, FD, WA-J: Design, Writing - Review & Editing, NT: Formal analysis, Writing - Original Draft; JH: Formal analysis, Software, Conceptualization, Writing - Original Draft, Supervision; MR: Design, Conceptualization, Project administration, Writing - Review & Editing. All authors contributed to the article and approved the submitted version.
